# Insecticidal Activity of the Essential Oils from Different Plants Against Three Stored-Product Insects

**DOI:** 10.1673/031.010.2101

**Published:** 2010-03-16

**Authors:** Abdurrahman Ayvaz, Osman Sagdic, Salih Karaborklu, Ismet Ozturk

**Affiliations:** ^1^ Erciyes University, Faculty of Arts and Sciences, Department of Biology, 38039, Kayseri, Turkey; ^2^ Erciyes University, Collage of Engineering, Food Engineering Department, 38039, Kayseri, Turkey; ^3^ Osmaniye Korkut Ata University, Faculty of Arts and Sciences, Department of Biology, 80020, Osmaniye, Turkey

**Keywords:** Botanical based fumigants, stored product insect management, savory, myrtle, oregano

## Abstract

This study was conducted to determine the insecticidal activity of essential oils from oregano, *Origanum onites* L. (Lamiales: Lamiaceae), savory, *Satureja thymbra* L. (Lamiales: Lamiaceae), and myrtle, *Myrtus communis* L. (Rosales: Myrtaceae) against three stored-product insects. Essential oils from three species of plants were obtained by Clevenger-type water distillation. The major compounds in these essential oils were identified using gas chromatography-mass spectrometry and their insecticidal activity was tested against adults of the Mediterranean flour moth *Ephestia kuehniella* Zeller (Lepidoptera: Pyralidae), the Indian meal moth *Plodia interpunctella* Hübner (Lepidoptera: Pyralidae) and the bean weevil *Acanthoscelides obtectus* Say (Coleoptera: Bruchidae). While the major compound found in oregano and savory was carvacrol, the main constituent of the myrtle was linalool. Among the tested insects, *A. obtectus* was the most tolerant species against the essential oils. However, the insecticidal activity of the myrtle oil was more pronounced than other oils tested against *A. obtectus* adults. The essential oils of oregano and savory were highly effective against *P. interpunctella* and *E. kuehniella,* with 100% mortality obtained after 24 h at 9 and 25 µl/l air for *P. interpunctella* and *E. kuehniella,* respectively. LC_50_ and LC_99_ values of each essential oil were estimated for each insect species.

## Introduction

The bean weevil, *Acanthoscelides obtectus* (Say) (Coleoptera: Bruchidae) is one of the most damaging pests of the kidney bean, Phaseolus vulgaris L. (Fabales: Fabaceae) in the Mediterranean area. It causes losses of up to 30% of stored beans (Pemonge et al. 1997). Its oviposition and growth are continuous, and the larvae feed on the seeds. After emergence from the seeds, the adults reproduce either in the field or in the stored seeds in a continuous cycle (Labeyrie 1962).

The Mediterranean flour moth, *Ephestia kuehniella* Zeller (Lepidoptera: Pyralidae), is one of the major pests in industrial flour mills in temperate climates (Jacob and Cox 1977). Larvae reduce product quality by their presence and by the production of frass and webbing, and they also cause direct damage by feeding (Johnson et al. 1997). The Indian meal moth, *Plodia interpunctella* (Hübner) (Lepidoptera: Pyralidae), is a cosmopolitan pest that infests a wide range of stored products including nuts, beans, processed foods and dried fruits (Simmons and Nelson 1975). The control of these pests in storage systems mainly depends on fumigants such as methyl bromide or phosphine. However, methyl bromide was banned in many countries starting in 2004 because of its ozone depleting properties (Hansen and Jensen 2002). Many alternatives have been tested to replace methyl bromide fumigation for stored-product and quarantine uses. There is an urgent need to develop safe alternatives that have the potential to replace the toxic fumigants, yet are effective, economical and convenient to use (Ayvaz et al. 2008). Many spices and herbs, and their extracts, are known to possess insecticidal properties that are frequently present in the essential oil fraction (Brattsten 1983; Schmidt et al. 1991; Shaaya et al. 1991). Most of the essential oil constituents are monoterpenoids, which are secondary plant chemicals and considered to be of little metabolic importance. The toxicity of a large number of essential oils and their constituents have been evaluated against a number of stored-product insects.

Over the past 15 years, interest in botanical insecticides has increased as a result of environmental concerns and insect populations becoming resistant to conventional chemicals. Botanical insecticides are naturally occurring insecticides that are derived from plants (Isman 2000). The insecticidal activity of essential oils and plant extracts against different stored-product pests has been evaluated (Shaaya et al. 1991; Sarac and Tunc 1995; Tunc et al. 2000; Kim et al. 2003; Lee et al. 2003; Asian et al. 2005; Cetin and Yanikoglu 2006; Negahban et al. 2007, Ayvaz et al. 2009). In spite of the wide-spread recognition that many plants possess insecticidal properties, only a handful of pest control products directly obtained from plants are in use because the commercialization of new botanicals can be hindered by a number of issues (Isman 1997). Botanicals used as insecticides presently constitute 1% of the world insecticide market (Rozman et al. 2007). Essential oils from different plant species possess ovicidal, larvicidal, and repellent properties against various insect species and are regarded as environmentally compatible pesticides (Isman 2000; Cetin et al. 2004).

In the present study, the chemical constituents of essential oils from *Satureja thymbra* L. (Lamiales: Lamiaceae), *Origanum onites* L. (Lamiales: Lamiaceae), and *Myrtus communis* L. (Rosales: Myrtaceae) were determined, and the insecticidal activity of these essential oils was tested against the adult stages of the stored-products pests: *E. kuehniella, P. interpunctella* and *A. obtectus.* No study has been reported previously concerning the activity of these compounds as fumigants against these stored product insects. The essential oils were applied primarily on adults to prevent mass egg production and further damages from larvae.

## Materials and Methods

### Insect cultures

The founding insect culture of *A. obtectus* was collected in infested kidney beans (*P. vulgaris*) that were stored in 5-liter plastic containers at a storehouse from Kayseri Province of Turkey. Adults oviposited on *P. vulgaris* beans, and the larvae developed inside the beans until adult emergence in 1-liter glass jars. To allow air passage, a hole 2 cm in diameter was opened in the center of each jar lid, and a sterile cloth was glued to the underside of each lid.

The Mediterranean flour moth, *E. kuehniella,* culture was obtained from the Department of Plant Protection, Faculty of Agriculture of Ankara University. *E. kuehniella* larvae were reared using a mixture consisting of one kg wheat flour, 55 g yeast and 30 g germs of wheat (Marec et al. 1999).

The Indian meal moths, *P. interpunctella,* used in this experiment were taken as larvae from naturally infested dried apricot collected from Kayseri province. The larvae of *P. interpunctella* were maintained continuously on a diet containing 10% glycerol, 50% dried apricot and 40% wheat flour with wheat bran mixture. Throughout the experiments insect cultures were maintained at constant temperature (27 ± 1°C), photoperiod (14L:10D) and relative humidity (60% ± 5) (Ayvez et al. 2007, 2009).

### Plant materials

The plants savory (*S. thymbra*) and myrtle (*M. communis*) were collected in the middle of July 2007 from the fields of Mersin (Southern Turkey), and oregano (*O. onites*) was collected in the middle of August 2007 from the fields of Canakkale (Western Turkey). Leaves were randomly collected from plant parts and shade dried.

### Extraction of essential oil

The plant leaves were air dried at room temperature and chopped into small pieces using a mill with rotary knives. The essential oil was extracted from the plants using a Clevenger-type water steam distillation apparatus (Papachristos and Stamopoulos 2004). The distilled essential oils were stored in a refrigerator at 4°C until being used in the treatments.

### Analysis of volatile compounds

The composition of the volatile constituents was established by gas chromatography-mass spectrometry/Quadropole detector analyses using a Shimadzu QP 5050 system fitted with a Free Fatty Acid Phase (50 m × 0.32 mm (i.d.) film thickness: 0.25 µm) capillary column. Detector and injector temperature were set at 230°C. The temperature program for the column was from 120°C (1 min) to 230°C at a rate of 6°C/min and than held at 200°C for 35 min. Helium was used as a carrier gas at a flow of 14 psi (Split 1:10), and the injection volume of each sample was one µl. The identification of the components was based on comparison of their mass spectra with those of Wiley and Nist Tutore Libraries (Adams 2001). The ionization energy was set at 70 eV.

### Insecticidal activity

In order to test the toxicity of essential oils on the adults (< 24 h age) of *E. kuehniella* and *P. interpunctella,* ten adults were put into the 1000 ml glass jars. Essential oils were applied on a filter-paper strip measuring 3 × 3 cm that was attached to the lower side of the jar's lid. Doses were calculated based on nominal concentrations and assumed 100% volatilization of the oils in the exposure vessels (Papachristos and Stamopoulos 2004). The same procedure was applied for the adults (< 48 h age) of *A. obtectus,* but this species was provided with kidney beans as food. All the insecticidal activity experiments were conducted at constant temperature (27 ± 1°C), photoperiod (14L: 10D) and relative humidity (60% ± 5). The adults of *E. kuehniella* and *P. interpunctella* were exposed to essential oil vapors (1.5; 3; 6; 9; and 25 µl/l air) for 24 h. However, because of high tolerance of *A. obtectus* adults were treated with higher doses (65; 130; and 195 µl/l air) and longer exposure time (24, 72 and 144 h) than that of *E. kuehniella* and *P. interpunctella.* A dose-mortality line depending on the exposure time(s) was developed, and the lethal concentration of essential oil needed to kill 50 or 99% of the pest population (LC_50_ and LC_99_, respectively) was determined. Three replicates were set up for each dose and exposure time. A complete set of controls was maintained and replicated three times for each treatment. All replicates ran simultaneously during the experiments.

### Statistical analysis

The data were corrected using Abbott's formula (Abbot 1925) for the mortalities in the controls, and the data were subjected to probit analyses using SPSS (2001) for Windows to estimate LC_50_ and LC_99_ values of the essential oils against each stored-product insect species. Percentage mortality values for different exposure times were subjected to analysis of variance (one-way ANOVA) using the same statistical program (SPSS 2001) for probit analysis. Data were transformed using arcsine √x transformation to meet normality, which is recommended for analysis of variance (ANOVA) (Steel and Torrie 1980). Means were separated at the 5% significance level by the least significant difference (LSD) test.

## Results

### Chemical composition of essential oils

The chemical constituents of three different essential oils are given in Table 1. The results of analysis showed that the chemical contents of oregano and savory were similar, but myrtle had a different chemical composition. The main compounds found in the savory were characterized as carvacrol (53.7%), γ -terpinen (17.6%), thymol (13%) and *p-*cymene (10.1%). Of all the compounds found in the oregano, the percentage of the carvacrol was the highest (70.3%) as in the savory. The main other compounds were linalool (11.9%) and thymol (9.3%). The chemical constituents of the myrtle were different from other oils tested, and the main components were linalool (31.3%), linalyl acetate (17.8%), and 1.8-cineole (14.7%). The other constituents of the savory, oregano and myrtle are given in Table 1.

### Insecticidal activity of essential oils against the *E. kuehniella* adults

**Figure 1.**
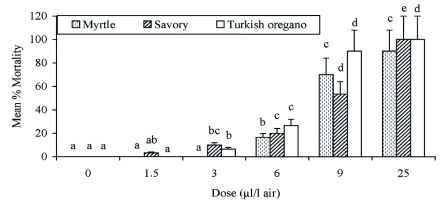
Percent mortality of the /Ephistia kuehniella/ adults after exposure to three different essential oils. Letters above bars indicate significant differences between doses. Bars with the same letter are not significantly different. Error bars indicate SD of means.High quality figures are available online.

The increasing doses of essential oils caused a significant increase in the mortality when the *E. kuehniella* adults were exposed to these oils for 24 h (for myrtle: *F* = 55.98; d.f. = 5, 12; p < 0.0001; for savory: *F* = 23.90; d.f. = 5, 12; p < 0.0001; and for oregano: *F* = 80.08; d.f. = 5, 12; p < 0.0001). Percent mortality of *E. kuehniella* was 90% at the 25 µl/l air dose when exposed to myrtle, and the same dose caused complete mortality (100%) when exposed to savory and to oregano (Figure 1).

**Table 1.**
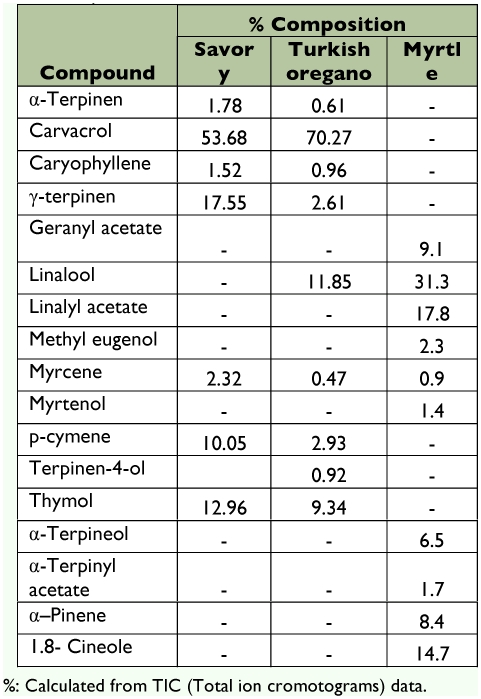
Chemical constituent of the essential oils obtained from three plants.

Probit analysis showed that LC_50_ values of these essential oils against *E. kuehniella* were 7.52, 10.34 and 12.74 µ l/l air for oregano, savory and myrtle, respectively. LC_99_ values revealed that *E. kuehniella* was more susceptible to oregano (12.72 µl/l air) than savory and myrtle (21.27 and 29.43 µl/l air for savory and myrtle, respectively) (Table 2).

### Insecticidal activity of essential oils against the *P. interpunctella* adults

**Figure 2.**
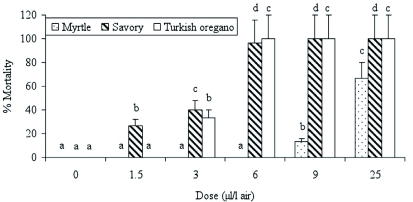
Percent mortality of the *Plodia interpunctella* adults after exposure to three different essential oils. Letters above bars indicate significant differences between doses. Bars with the same letter are not significantly different. Error bars indicate SD of means. High quality figures are available online.

The mortality values significantly increased depending on the increasing essential oil concentration when the *P. interpunctella* adults were exposed to myrtle, savory and oregano (for myrtle: *F* = 135.32; d.f. = 5, 12; p < 0.0001; for savory: *F* = 067.73; d.f. = 5, 12; p ;< 0.0001; and for oregano: *F* = 100.48; d.f. = 5, 12; p < 0.0001). The mortality values reached 96.7 and 100% when the adults were exposed to 6 µ l/l air concentrations of savory and oregano, respectively, and all the adults were killed by a 9 µ l/l air or higher concentration (Figure 2). The mortality effect of myrtle was lower than those of the other oils, and the rate of adult mortality was 66.7% at the 25 µ l/l air myrtle concentration. LC_50_ and LC_99_ values of the essential oils tested are summarized in Table 2, and these values revealed that the most effective essential oil against *P. interpunctella* adults was oregano, the same as for *E. kuehniella.* LC_50_ and LC_99_ values of the oregano against *P. interpunctella* were 4.06 and 5.77 µ l/l air, respectively (Table 2).

**Table 2:**
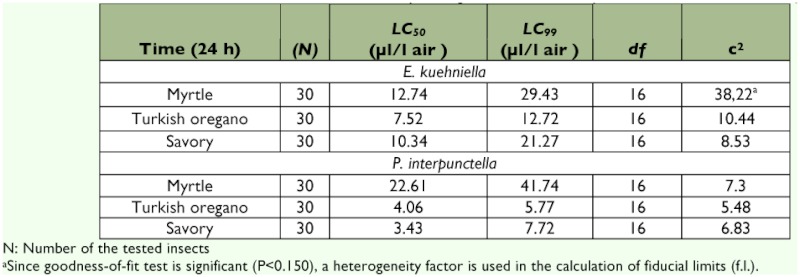
LC_50_ and LC_99_ values of essential oils from different plants against the adults of *Ephisitia kuehniella* and *Plodia interpunctella*

### Insecticidal activity of essential oils against the *A. obtectus* adults

**Figure 3.**
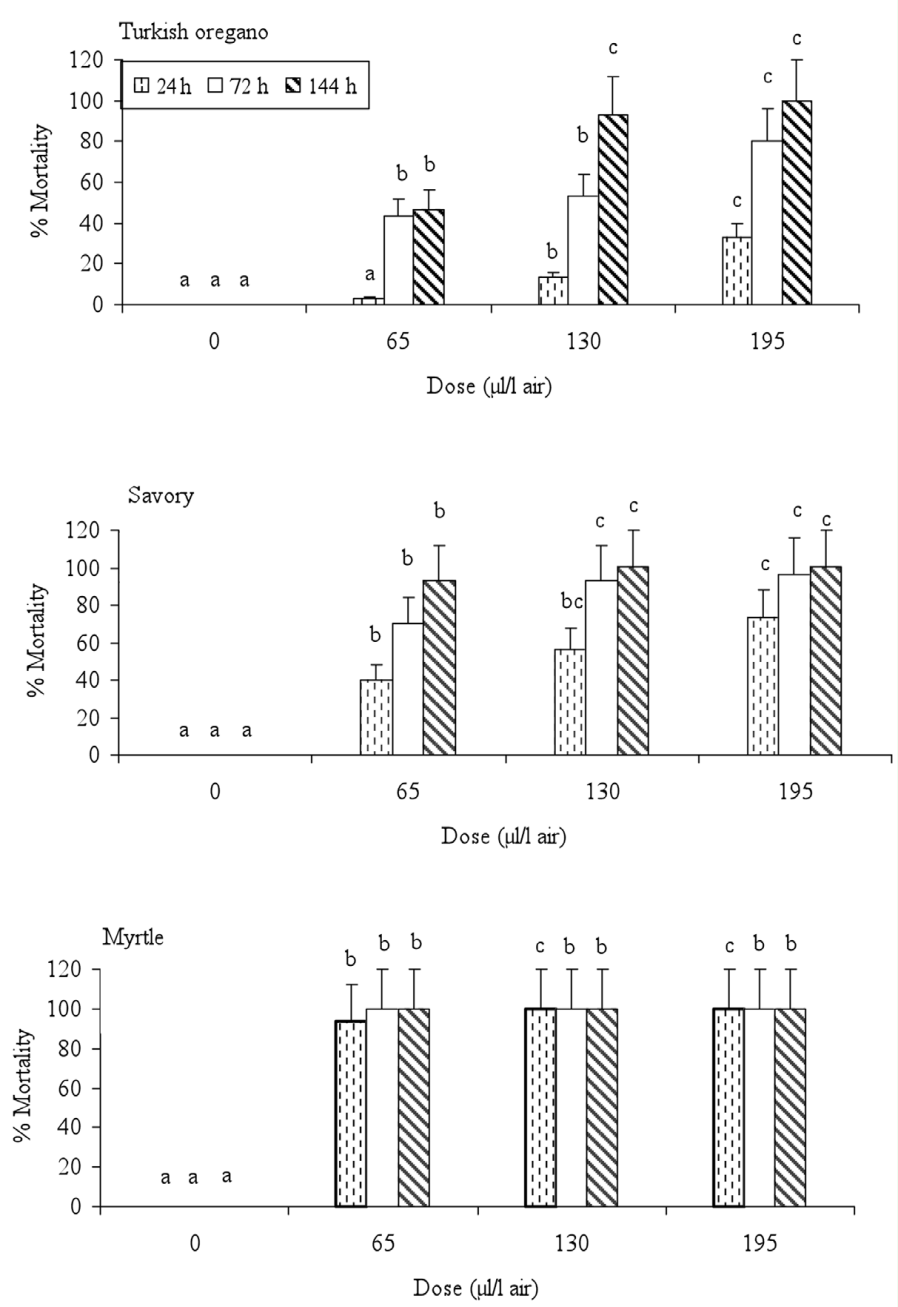
Percent mortality of the *Acanthoscelides obtectus* adults after exposure to three different essential oils. Letters above bars indicate significant differences between doses. Bars with the same letter are not significantly different. Error bars indicate SD of means. High quality figures are available online.

Among the tested insects, *A. obtectus* was the most tolerant species against the essential oils. To kill the adults of this insect by using these three essential oils, higher concentrations and exposure times are required than are required for the other two species. The concentration (25 µl/l air) that caused complete mortality for *E. kuehniella* and *P. interpunctella* did not affect *A. obtectus* adults. The myrtle was more effective than the other essential oils against *A. obtectus* adults. Increasing mortality was observed for the *A. obtectus* adults when the concentrations and exposure times increased. Although 100% mortality was obtained after 144 h at 195 µl/l air when *A. obtectus* was exposed to oregano and savory, the myrtle showed the same effect at 65 µl/l air and 72 h of exposure (Figure 3).

LC_50_ and LC_99_ values of the essential oils tested for the length of the time are summarized in Table 3, and the LC_50_ and LC_99_ values of the myrtle against *A.* *obtectus* were 33.56 and 50.97 µl/l air, respectively, for the highest exposure time. The doses required for 99% mortality (LC_99_) for using oregano and savory against *A. obtectus* were 127.36 and 76.05 µ l/l for the 144 h exposure, respectively (Table 3).

## Discussion

The insecticidal constituents of many plant extracts and essential oils are monoterpenoids. Due to their high volatility they have fumigant activity that might be of importance for controlling stored-product insects (Konstantopoulou et al. 1992; Regnault-Roger and Hamraoui 1995; Ahn et al. 1998). In the current study, the essential oils obtained from savory, oregano and myrtle showed insecticidal activity against the adults of *E. kuehniella, P. interpunctella* and *A. obtectus. A. obtectus* was the most tolerant species against these oils as the doses required to kill the adults of this species were much higher than those required for the other two pest species for all treatments. The adults of *A. obtectus* were more susceptible to myrtle oil than oregano or savory. The toxic effects of the myrtle could be attributed to major constituents such as linalool (31.3%), linalyl acetate (17.8%) and 1.8-cineole (14.7%). The high toxicity of linalool, linalyl acetate and 1.8-cineole was reported against the rice weevil *Sitophilus oryzae* and *Rhyzopertha dominica* (Rozman et al. 2007). Due to the linalool, linalyl acetate and 1.8-cineole constituents, the myrtle could also be used effectively against *S. oryzae* and *R. dominica.* These results, and those reported earlier, indicate that the insecticidal activity of the essential oils varies depending on the stage of the insect, the species and the plant origin of the essential oil (Tunc et al. 2000; Chiasson et al. 2001; Choi et al. 2003; Sedy and Koschier 2003; Negahban et al. 2007).

The essential oils of oregano and savory were highly effective against *P. interpunctella* and *E. kuehniella,* and 100%) mortality was obtained after 24 h at 9 and 25 µl/l air for these species, respectively. The major components of these two essential oils are monoterpenes, primarily carvacrol and thymol. Reported biological activities of plant terpenoids include repellency and deterrence, reduced palatability, growth inhibition through altered protein availability, enzyme inhibition, and direct toxicity (Harborne 1993). The plants oregano and savory belong to the family Lamiaceae. Jacobson (1989) pointed out that the most promising botanical insect-control agents are in the families of Annonaceae, Asteraceae, Canellaceae, Lamiaceae, Meliaceae and Rutaceae.

The insecticidal activity against *A. obtectus* of the essential oils obtained from oregano and savory was higher at the lowest dose and longest exposure period than at the highest dose and the lowest exposure period (Figure 3). El-Nahal et al. (1989) stated that the period of exposure appears to be more important than dosage in affecting the efficiency of the vapors of *Acorus calamus* essential oil to adults of five stored-product insect species. Similar results have been reported for the toxicity of methanol extract of the rhizome from *Acorus gramineus* to adults of *S. oryzae* and *Lasioderma serricorne* (Park 2000).

Similarly, the essential oils of oregano and savory have been found to be lethal to *P. interpunctella* and *E. kuehniella* but less toxic to *A. obtectus.* Carvacrol and thymol are usually extracted from *Satureja, Coridothymus, Thymbra,* and *Origanum* species and have been found to be lethal to turnip aphids (Chiasson et al. 2001). There are numerous reports on the insecticidal activity of the essential oils from *Origanum* species, and the major components of this species, such as carvacrol, thymol, γ-terpinen and terpinen-4-ol, are based on fumigant and repellent activity rather than contact toxicity (Tunc et al. 2000; Erler and Tunc 2005; Erler 2005).

**Table 3:**
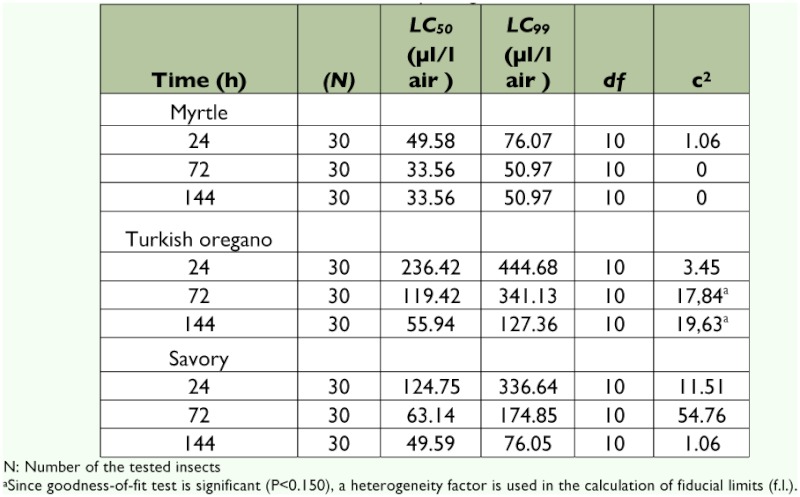
LC_50_ and LC_99_ values of essential oils from different plants against the adults of *Acanthoscelides obtectus*

Carvacrol has broad insecticidal and acaricidal activity against agricultural, stored-product and medical pests, and it acts as a fumigant. It is highly toxic to nymphs of the termite *Reticulitermes speratus,* adults of the rice weevil *S. oryzae,* the pulse beetle *Callosobruchus chinensis,* the cigarette beetle, *L. serricorne* (Ahn et al. 1998), and the mite, *Tetranychus urticae* (Isman 2000).

The insecticidal activity of the myrtle was lower than that of the oregano and savory against *E. kuehniella* and *P. interpunctella.* The percent mortality of both moth adults was 100% at the 25 µl/l air and 24 h exposure when treated with oregano and savory. However, percent mortality of *E. kuehniella* and *P. interpunctella* was 90% and 67%, respectively, when exposed to myrtle.

If the cost-effective commercial problems can be solved, the essential oils obtained from these plants can be effectively used as part of integrated pest management strategies (Rozman et al. 2007). The essential oil content of aromatic plants is about 1–3% (Cakir 1992). Therefore large quantities of plant material have to be processed in order to obtain the essential oils in quantities sufficient for commercial-scale tests (Tunc et al. 2000). It would be useful to breed the plants containing desired essential oils in elevated quantity.

The high activity of these compounds could make it a potential substitute for methyl bromide in various uses in stored-product control programs and can be used in coordination with microbial insecticides, attractants and traps, and beneficial insects and mites (natural enemies of pests) as a component of the integrated pest management. The observed fumigant activity shows that essential oils are sources of biologically active vapors that are potentially efficient insecticides. Consequently, the possibility of employing these natural fumigants to control insects in stored products may be worthy of further investigation.
